# Systematic Review of Intravenous Ceftriaxone Administration in Animals

**DOI:** 10.1002/vms3.71027

**Published:** 2026-06-23

**Authors:** Paula Maria Fernandes de Vasconcelos, Gabriel de Brito Rodrigues, Michel Leandro de Campos

**Affiliations:** ^1^ Instituto de Ciências da Saúde Universidade Federal de Mato Grosso Sinop Mato Grosso Brazil

**Keywords:** animal, antibiotic, ceftriaxone, pharmacokinetics, pharmacology

## Abstract

**Background:**

Ceftriaxone is an antibiotic frequently prescribed in veterinary medical practice due to its effectiveness against several pathogenic microorganisms responsible for diseases in different animal species.

**Objective:**

To select scientific articles from the academic databases PubMed and Web of Science that analysed the pharmacokinetic aspects of intravenously administered ceftriaxone in animals.

**Methods:**

In accordance with the principles of scientific transparency, the systematic review protocol and all research stages were previously registered on the Open Science Framework (OSF) platform. The scientific articles that met the inclusion criteria were subjected to a checklist comprising 24 minimum assessment items designed for reporting pharmacokinetic studies.

**Results:**

A total of 52 pharmacokinetic profiles were identified from the 29 selected scientific articles, including records involving 13 different animal species used in ceftriaxone experimental studies.

**Conclusion:**

The limitations and inconsistencies identified in this review highlight the importance of detailed reporting of pharmacokinetic data derived from experimental trials in animals.

## 1 Introduction

Ceftriaxone is listed by the World Health Organization as one of the essential medicines recommended for the treatment of infections caused by microorganisms prone to resistance against other antibiotics (World Health Organization 2023). From a pharmacodynamic perspective, ceftriaxone exerts its action by binding to bacterial cell wall proteins, thereby disrupting the third and final stage of the bacterial cell wall (Mora‐Ochomogo and Lohans 2021). Its effectiveness stems from its broad‐spectrum activity against gram‐positive bacteria (*Steptococcuss* spp. and *Staphylococcus* spp.) (Nazir et al. 2025), gram‐negative bacteria (*Escherichia coli*, *Proteus* spp., *Klebsiella* spp., *Enterobacter* spp., *Salmonella* spp. and *Pasteurella* spp.) (Rodríguez‐Baño et al. 2018; Tamma et al. 2023) and certain anaerobic bacteria (*Bacteroides fragilis*, *Peptostreptococcus* spp., Clostridium spp.) (Ligero‐López et al. 2023).

Experimental evidence has demonstrated that ceftriaxone promotes a reduction in inflammation and airway resistance in septic rats, although it is associated with pharmacokinetic alterations characterized by decreased bioavailability and increased clearance (Simões et al. 2024). Additionally, the investigation of the kinetic disposition and efficacy of ceftriaxone in cows with staphylococcal mastitis allowed the identification of therapeutic concentrations of the drug and its circulating metabolite, which were associated with the elimination of *Staphylococcus aureus* and the clinical resolution of the disease (Buragohain et al. 2021). However, the pharmacokinetic evaluation of ceftriaxone and meropenem in dogs indicated efficacy against susceptible bacteria, with meropenem demonstrating superior effectiveness compared to ceftriaxone (Orooba and Shwaish 2024).

The pharmacokinetics of ceftriaxone provide crucial information regarding its disposition in the animal body, encompassing routes of administration, distribution, metabolism, excretion and potential toxicity (Orooba and Shwaish 2024). Among the available administration routes, intravenous injection is recognized for achieving high therapeutic efficacy and extensive distribution across most tissues and body fluids in animals (Al Nasr et al. 2025). Additionally, the affinity of ceftriaxone for plasma protein binding indicates that higher binding reduces the rate of drug transfer between plasma and extravascular compartments, contributing to its prolonged elimination half‐life (Ahmed et al. 2025). Moreover, the free fraction of the drug requires sufficiently high systemic concentrations to attain the desired therapeutic effect (Gonzalez et al. 2013).

Accordingly, the administration of ceftriaxone demands a rational, scientifically grounded approach to dosing regimens across different species, as improper management may result in toxicity or therapeutic failure (Poapolathep et al. 2020). Therefore, the objective of this systematic review was to identify and analyse scientific articles from academic databases that reported pharmacokinetic (PK) parameters of intravenously administered ceftriaxone in animals.

## 2 Materials and Methods

### 2.1 Investigation Method

The first methodological procedure consists of identifying articles available on digital scientific publication platforms, such as PubMed and Web of Science. The search descriptors used in English were pharmacokinetics, ceftriaxone and animal, combined using the operators “AND” and “All Field” (Table 1).

**TABLE 1:** Complete search plan in the digital platform databases.

Database	Applied descriptors
**PubMed**	((pharmacokinetics) AND (ceftriaxone) AND (animal))
**Web of Science**	pharmacokinetics (All Fields) and ceftriaxone (All Fields) and animal (All Fields)

During this exploration phase, platform‐specific filtering tools, such as publication date or type, were not applied. In line with scientific transparency principles, the protocols for this systematic review were preregistered on the Open Science Framework (OSF) and are available at: osf.io/bsn74.

### 2.2 Selection Criteria

The selection of scientific articles from PubMed and Web of Science included in this systematic review followed established methodological criteria (Okoli and Duarte 2019). Based on this approach, the titles and abstracts retrieved from the databases were initially screened for eligibility. To enhance efficiency, the virtual platform Rayyan was used to manage and coordinate study selection, allowing collaborative review among team members.

Screening was conducted independently and first by two reviewers, while a third reviewer was consulted in case of discrepancies. This phase was developed by applying exclusion criteria that omitted articles written in non‐Roman scripts, languages other than English or Portuguese, studies on the pharmacological associations of ceftriaxone, literature reviews, non‐intravenous administration routes and studies that did not provide comprehensible pharmacokinetic (PK) parameters of ceftriaxone obtained from animal experiments.

Following initial screening, the remaining articles were fully assessed to select studies reporting the pharmacokinetics of intravenously administered ceftriaxone in healthy and diseased animals, irrespective of sex. Selected studies were required to report at least key PK parameters, including elimination half‐life, elimination rate constant, clearance, area under the curve (AUC) and plasma free fraction or concentration. All selection steps were documented in a PRISMA 2020 flowchart (Galvão et al. 2015) to ensure full traceability of decisions throughout the review.

### 2.3 Analysis of the Integrity of Selected Scientific Articles

The scientific articles that met the inclusion criteria of this review were evaluated using the ClinPK questionnaire (Kanji et al. 2015), which consists of a 24‐item checklist designed to verify the minimum essential criteria for conducting pharmacokinetic studies, in line with PRISMA guidelines for systematic reviews. During the application of this method, items 12, 14, 20 and 21 were disregarded, as they were not applicable to animal study designs.

The presentation of results from the checklist was systematized using an adaptation of the Evidence Gap Maps (EGM) methodology (Campbell et al. 2023). The procedure enhanced transparency and reliability by identifying reporting gaps, classifying studies by checklist adherence (100%, 50%, 0%), and ensuring clear and consistent analysis of ceftriaxone data across animal species.

### 2.4 Systematic Assessment of Pharmacokinetic Parameters

The data extraction procedure comprised the identification of PK parameters reported in the studies included in the review, followed by verification of the availability of corresponding mean values and standard deviation (SD) indicators associated with these parameters. The standardization of measurement units for ceftriaxone PK data was conducted using unit conversion established in the literature (Ogden and Fluharty 2015; Ansel et al. 2016; Bauer 2014; Maradiya et al. 2010), ensuring consistency with the intrinsic mathematical relationships governing each extracted PK parameter.

Among the mean values and corresponding SDs of the ceftriaxone PK parameter reported in the selected articles, the following were included in this review: AUC from zero to the last observation (AUC_0‐t_), AUC from zero to infinity (AUC_0‐∞_), clearance (Cl), elimination rate constant (Kel or β), free fraction (FU), elimination half‐life (T_1/2_) and volume of distribution (V_d_). Furthermore, compartmental modelling data reported in the studies were organized according to the number of compartments, parameter type and animal species investigated.

The final methodological step for PK parameter extraction consisted of systematically compiling an electronic spreadsheet, which included additional information on the selected experimental trials, such as administered doses (mg kg^−1^), animal species, sex (female or male), age (years), weight (kg), number of animals and pharmacokinetic profile. Additionally, this information verified whether the studies monitored ceftriaxone administration alone or in combination with another drug, as well as the use of different doses and the clinical status of the animals. Thus, the systematization of parameters considering these informative elements was implemented to support the interpretative analysis of the experimental design of ceftriaxone administration.

## 3 Results and Discussion

### 3.1 Investigation and Selection

**FIGURE 1:**
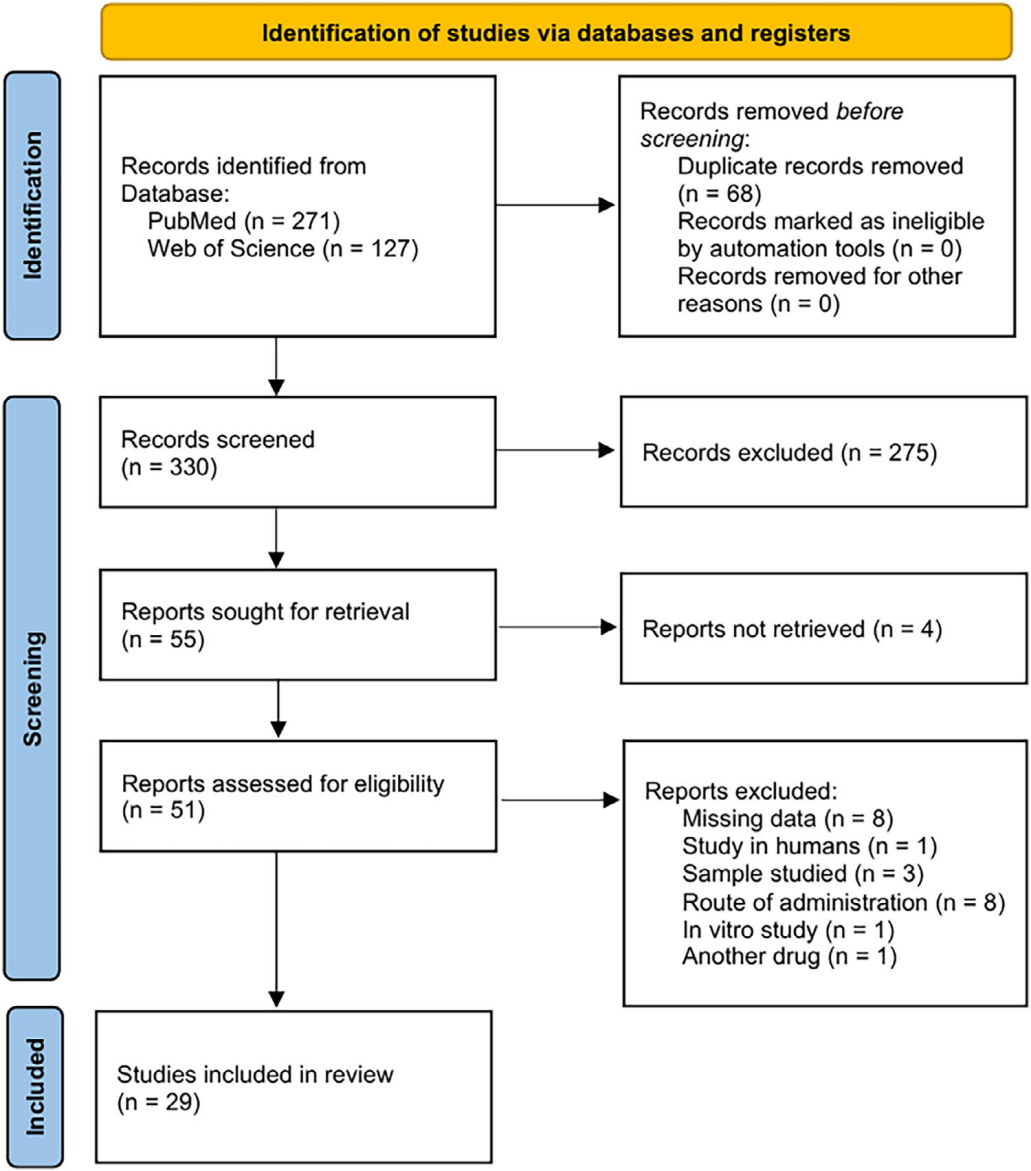
Flowchart of the systematic review of ceftriaxone.

The investigation conducted in the databases resulted in a total of 398 scientific articles, of which 271 studies were registered in PubMed and 127 studies in Web of Science. The subsequent phase consisted of the initial screening of 330 articles using the digital platform Rayyan, leading to the identification of 55 studies that met the selection criteria established at this screening stage. Ultimately, the full‐text evaluation performed on 51 articles accessible in the databases identified 29 studies that adequately fulfilled all previously defined selection criteria (Figure 1).

Considering the commitment to accuracy of the results obtained during the stages of applying the selection criteria to the scientific articles identified in the previously established platforms, it is important to note that the incorporation of the digital tool Rayyan was decisive for the identification and removal of 68 duplicate records, followed by the elimination of a total of 275 publications during the initial screening that met the exclusion criteria. In the final screening stage, four studies were found to have no full‐text access available, while full‐text assessment resulted in the exclusion of 22 articles for not meeting the eligibility criteria, yielding a total of 29 studies included in this review on the pharmacokinetics of intravenously administered ceftriaxone in animals.

### 3.2 Study Characteristics

The systematic survey of information provided by the selected articles contributed to the identification of 52 pharmacokinetic profiles from 13 different animal species that received ceftriaxone in their experiments (Table 2). A detailed evaluation of these studies revealed 42 profiles (80.77%) from healthy animals, whereas the remaining 10 profiles (19.23%) were derived from animals with specific clinical conditions, such as prematurity, experimentally induced febrile state, meningitis, endometritis, hepatopathy and nephropathy in the species investigated. Regarding the temporal distribution of the selected articles, 6 studies were published in the 1980s, followed by 7 studies in the 1990s, and from the 2000s onwards, 16 studies were made available presenting PK data related to ceftriaxone administered in animals.

**TABLE 2:** Baseline information extracted from selected articles.

References	Investigated species	No. of animals	No. of PK profiles	Sex	Age (years)	Weight (kg)
Albarellos et al. (2007)	Cats mixed breeds	5	1	3 F		5.51 ± 1.83
				2 M		
Cavalier et al. (1997)	Yucatan miniature pigs	3	2^a^, ^b^	F	1	33.5 ± 3.6
Cetin et al. (2021)	Akkaraman sheep	8	3^a^	F	2.4 ± 0.3	44 ± 4
Corum et al. (2019)	Holstein Calves	6	1	3 F	Premature	25.04
				3 M		
Dardi et al. (2005)	Buffalo calves	5	1	M	0.88–1	95
Dhanani et al. (2020)	Merino sheep	8	3^a^, ^c^	M	3–4	36.5–49.2
Gardner et al. (1994)	Mixed breeds mares	6	1	F		361–541
Gohil et al. (2009)	Bubalus bubalis	6	1	M	0.75–0.83	70–100
Goudah et al. (2006)	Ewes	6	1	F	2–3	40–47
Goudah (2008)	Camelus Dromedarius	5	1	F	6–9	350–490
Guerrini et al. (1985)	Merino ewes	6	1	—	2–3	42.6 ± 2.9
Hakim et al. (1989)	Wistar rats	12	1	M	Mature	0.285 ± 0.027
Ismail (2005)	Goat	10	1	F	2–2.5	25–30
Johal et al. (1999)	Crossbred calves	12	1	M	1–1.5	60–175
Kovar et al. (1997)	Wistar rats	10	2^b^	M		0.26–0.31
Kwon et al. (1985)	Australian original white rabbits	18	2^a^	M	0.67–0.83	2.6–3.2
Kwon and Bourne (1986)	Wistar rats	12	3^a^	M		0.22–0.26
Kumar et al. (2010)	Crossbred Cows	16	2^c^	F	3–9	
Lutsar et al. (1997)	New Zealand White rabbits	28	4^b^, ^c^	M		2–2.2
Sar et al. (2008)	Goats Black Bengal	18	3^c^	F	1.5–2	10–14
Mapongpeng et al. (2019)	Chelonia mydas	5	2^b^		2–3	15.02 ± 1.56
Maradiya et al. (2010)	Crossbred calves	6	1	M	0.5–0.75	55–105
Matsui et al. (1984)	Beagle	3	1	3 F		12–12.6
	Rhesus	2	1	2 M		5.3–5.9
Ranjan et al. (2011)	Chotanagpuri sheep	12	2^c^	F	2–2.5	13–15
Rebuelto et al. (2002)	Mixed breed dogs	6	1	3 F		19.6 ± 2.2
				3 M		
Ringger et al. (1996)	Horses	4	1	2F		305–559
	Poney	1		3 M		
Ringger et al. (1998)	Thoroughbred foals	5	1		2–12 days	60–79.5
Soback and Ziv (1988)	Calves (Israeli‐Friesian)	10	1	M	0.04–0.08	
Zhang et al. (2019)	Sprague Dawley rats	36	9^a^ *^,^* ^b^	M	0.83	0.526–0.046

*Note*: Sex of the species: male (M) and female (F).

^a^ Ceftriaxone isolated versus coadministration with other drugs.

^b^ Administration of ceftriaxone at different doses.

^c^ Healthy animals versus clinical condition of the animals (febrile state, meningitis, endometritis, hepatopathy and nephropathy).

The compiled data demonstrated ceftriaxone pharmacokinetic parameters across several species, totalling 284 animals (Table 3): 44 (15.49%) cattle, 11 (3.87%) buffaloes (*Bubalus bubalis*), 28 (9.86%) goats, 9 (3.17%) dogs, 5 (1.76%) camels (*Camelus dromedarius*), 16 (5.63%) horses, 46 (16.20%) white rabbits, 5 (1.76%) cats, 2 (0.70%) monkeys, 3 (1.06%) miniature pigs (*Yucatan miniature pigs*), 40 (14.08%) sheep, 70 (24.65%) rats and 5 (1.76%) green turtles (*Chelonia mydas*). Profile analysis of these animals revealed particularities among individuals of the same species, such as differences in body proportions between horses and ponies. Some studies also showed divergences regarding the breeds investigated, as observed in sheep (*Akkaraman sheep*; *Merino sheep*; *Chhotanagpuri sheep*) (Cetin et al. 2021; Dhanani et al. 2020; Ranjan et al. 2011) and calves (*Crossbred calves*; *Holstein calves*; *Israeli‐Friesian calves*) (Corum et al. 2019; Johal and Srivastava 1999; Soback and Ziv 1988), whereas other studies included mixed‐breed animals, specifically in cats, dogs and mares. Additionally, lineage diversity was observed in laboratory animals, including regional lineages in white rabbits (*Australian original white rabbits* and *New Zealand white rabbits*) (Kwon et al. 1985; Lutsar et al. 1997) and laboratory strains in rats (*Sprague Dawley rats* and *Wistar rats*) (Hakim et al. 1989; Zhang et al. 2019).

**TABLE 3:** Data extracted using standardized units.

Reference	Covariates	Species	Dose	AUC_0‐t_ (ng · h mL^−1^)	AUC_0‐∞_ (ng · h mL^−1^)	Kel or β (h^−1^)	T_1/2_ (h)	Cl (L h^−1^)	V_d_ (L)	FU	V_1_ (L)
Albarello et al. (2007)		Cats mixed breeds	25 mg kg^−1^		77750 ± 30610	0.48 ± 0.10	1.73 ± 0.23	2.04 ± 0.72	3.14 ± 1.21		3.1407 ± 1.2122
Cavalier et al. (1997)		Yucatan miniature pigs	1 g 12 h kg^−1^		15200 ± 2250	0.63 ± 0.17	1.1 ± 0.3	6.65 ± 1.07			
Cetin et al. (2021)	Isolated		200 mg Day^−1^ kg^−1^		129200 ± 41200	0.77 ± 0.17	0.9 ± 0.2	8.21 ± 2.21			
	Ketoprofen (3 mg)	Akkaraman sheep	40 mg kg^−1^		182410 ± 20270	0.49 ± 0.04	1.42 ± 0.12	9,68 ± 0.88	7.48 ± 0.88	0.61 ± 0.55%	
	Acid tolfenamic (88 mg)				287970 ± 15700	0.33 ± 0.035	2.09 ± 0.22	6.16 ± 0.44	7.92 ± 0.88		
					256280 ± 21430	0.32 ± 0.026	2.18 ± 0.18	7.04 ± 0.44	7.04 ± 0.44		
Corum et al. (2019)	Premature	Holstein Calves	20 mg kg^−1^		174340 ± 40750	0.21 ± 0.02	3.31 ± 0.37	3 ± 0.75	11.2 ± 2.75		
Dardi et al. (2005)	Febrile	Buffalo calves	10 mg kg^−1^		25200 ± 1970	0.35 ± 0.02	2.04 ± 0.14	38.95 ± 2.85	114.95 ± 14.25		
Dhanani et al. (2020)	Normoalbuminemia and Ertapenem	Merino sheep	40 mg kg^−1^	83600 ± 13400		1.2 ± 0.4	0.58 ± 0.19	21.9 ± 3.1	19.9 ± 5.9	0.801 ± 0.037	
Dhanani et al. (2020)	Hypoalbuminemia and Ertapenem	Merino sheep	40 mg kg^−1^	65000 ± 13500		1,1 ± 0.2	0.63 ± 0.11	28.5 ± 5.1	26.8 ± 6.2		
	Hypoalbuminemia and Ertapenem			284400 ± 40100		0.7 ± 0.1	0.99 ± 0.14	6.4 ± 1	9.7 ± 1		
Gardner et al. (1994)		Mixed breeds mares	14 mg kg^−1^			0.89 ± 0.20	0.81 ± 0.16	76.37 ± 14.68	67.9 ± 9.06		45.3 ± 9.06
Gohil et al. (2009)		Bubalus bubalis	10 mg kg^−1^		40000 ± 4270	0.55 ± 0.02	1.27 ± 0.04	22.44 ± 2.24	30.6 ± 3.4		
Goudah et al. (2006)		Ewes	10 mg kg^−1^		77050 ± 12710	0.4 ± 0.05	1.75 ± 0.02	6.09 ± 4.35	12.1 ± 6.52	67	
Goudah (2008)		Camelus Dromedarius	10 mg kg^−1^		91870 ± 14310	0.27 ± 0.13	2.57 ± 0.52	46.2 ± 4.2	134. ± 4.2		
Guerrini et al. (1985)		Merino ewes	47 mg kg^−1^		211500 ± 65600	0.40 ± 0.24	1.73	9.37 ± 3.83	12.7 ± 4.69		
Hakim et al. (1989)		Wistar rats	100 mg kg^−1^	570000000 ± 144000000		1.5 ± 0.3	1710 ± 390	0.19 ± 0.05	0.02 ± 0.0034	78% ± 98%	45.3 ± 9.06
Ismail (2005)		Goat	20 mg kg^−1^		84200 ± 6300	0.48 ± 0.03	1.44 ± 0.01	6.52 ± 0.41	10.1 ± 0.52	61% ± 55%	3,41 ± 0,22
Johal et al. (1999)		Crossbred calves	10 mg kg^−1^		32630 ± 1470	0.172 ± 0.02	4.39 ± 0.63	36.42 ± 1.17	81.07 ± 8.22	61%	9.4 ± 0,3525
Kovar et al. (1997)		Wistar rats	50 mg kg^−1^		25300000 ± 7200000	0.38 ± 0.13	1.8 ± 0.6	0.03 ± 0.02	0.09 ± 0.05		0.04845 ± 0.00285
			100 mg kg^−1^		43500000 ± 8200000	0.33 ± 0.06	2.1 ± 0.4	0.04 ± 0.01	0.11 ± 0.02		0.05415
Kwon et al. (1985)	Isolated	Australian original white rabbits	30 mg kg^−1^		724000 ± 190000	1.31 ± 0.71	4.35 ± 2.17	0.11 ± 0.03	0.56 ± 0.33		0.1421 ± 0.1102
	Caffeine (5 mg kg^−1^)				949000 ± 229000	0.38 ± 0.17	3.38 ± 1.24	0.08 ± 0.02	0.37 ± 0.07		0.2813 ± 0.0957
	Caffeine (10 mg kg^−1^)				863000 ± 112000	0.38 ± 0.10	3.87 ± 1.04	0.09 ± 0.02	0.38 ± 0.08		0.2726 ± 0.1044
Kwon et al. (1985)	Isolated	Wistar rats	100 mg kg^−1^		612000	0.71	0.98	0.04		5.6 ± 32.8%	0.0104
	Caffeine (20 mg kg^−1^)				516000	0.89	0.78	0.05		5.5 ± 33.5%	0.011
Kumar et al. (2010)	Healthy	Crossbred Cows	6.72 mg kg^−1^		62200 ± 23300	0.70 ± 0.05	1.02 ± 0.07				
Kumar et al. (2010)	Endometrict	Crossbred Cows	6.72 mg kg^−1^		37000 ± 17100	0.52 ± 0.08	1.56 ± 0.25				
Lutsar et al. (1997)	Meningit	New Zealand White rabbits	150 mg kg^−1^	961000 ± 234000		0.26 ± 0.035	2.7 ± 0.36				
			200 mg kg^−1^	1393000 ± 273000		0.22 ± 0.079	3.07 ± 1.1				
			300 mg kg^−1^	1777000 ± 158000		0.28 ± 0.049	2.5 ± 0.44				
			400 mg kg^−1^	1150000 ± 146000		0.34 ± 0.013	2.06 ± 0.08				
Sar et al. (2008)	Health	Goats Black bengal	50 mg kg^−1^		31000 ± 5390	3.57 ± 0.04	0.19 ± 0.002	16.92 ± 2.88	5.64 ± 0.96		
	Hephatopatic				88730 ± 13850	1.80 ± 0.06	0.38 ± 0.01	6.96 ± 1.08	3.84 ± 0.48		
	Nephropatic				43030 ± 8670	2.10 ± 0.06	0.32 ± 0.008	12.48 ± 2.16	6.96 ± 1.2		
Mapongpeng et al. (2019)		Chelonia mydas	10 mg kg^−1^	70630 ± 13900	70990 ± 13970	0.23 ± 0.03	3.12 ± 0.51	2.18 ± 0.41	6.84 ± 1.45	80% ± 71%	
			25 mg kg^−1^	266500 ± 16790	267440 ± 17020	0.12 ± 0.02	5.81 ± 0.70	1.41 ± 0.09	7.83 ± 1.52		
Maradiya et al. (2010)		Crossbred calves	10 mg kg^−1^	56590 ± 6340	57350 ± 7040	0.44 ± 0.02	1.58 ± 0.06	15.12 ± 1.97	16 ± 2.4		45.3 ± 9.06
Matsui et al. (1984)		Beagle	20 mg kg^−1^		84300 ± 3900	0.82 ± 0.014	0.84 ± 0.015	2.94 ± 0.124	3 ± 0.19		1.6368 ± 0.124
		Rhesus			571000 ± 1105	1.54 ± 2.16	2.32 ± 4.48	0.101 ± 0.196	0.62 ± 0.63		0.4088 ± 0.504
Ranjan et al. (2011)	Healthy	Chotanagpuri sheep	50 mg kg^−1^		75710 ± 1660	1.17 ± 0.04	0.60 ± 0.02	9.47 ± 0.20	8.12 ± 0.14		
	Febrile				72260 ± 3100	0.76 ± 0.03	0.92 ± 0.03	9.65 ± 0.40	12.74 ± 0.42		
Rebuelto et al. (2002)		Mixed breed dogs	50 mg kg^−1^		240100 ± 55630	0.78 ± 0.03	0.88 ± 0.03	4.24 ± 0.92	5.43 ± 1.38		
Ringger et al. (1996)		Horses and Poney	50 mg kg^−1^		122212	0.43 ± 0.11	1.62 ± 0.42	135.09 ± 16.42	142.90 ± 5.10		
Ringger et al. (1998)		Thoroughbred foals	25 mg kg^−1^			0.39 ± 0.27	3.25 ± 3.22	40.11 ± 22.26	111.95 ± 75.54		
Soback and Ziv (1988)		Calves (Israeli‐Friesian)	10 mg kg^−1^		191580000 ± 27870000	0.49 ± 0.05	1.4 ± 0.14	0.19 ± 0.02	0.30 ± 0.05		
Zhang et al. (2019)	Isolated	Sprague Dawley rats	90 mg kg^−1^ day^−1^	531920 ± 27860	539310 ± 20520	0.46 ± 0.05	1.51 ± 0.18	0.09 ± 0.003	0.19 ± 0.16		
	Danhong			362540 ± 66100	363800 ± 66640	0.67 ± 0.24	1.04 ± 0.38	0.14 ± 0.019	0.20 ± 0.08		

^a^ Ceftriaxone isolated versus coadministration with other drugs.

^b^ Administration of ceftriaxone at different doses.

^c^ Healthy animals versus clinical condition of the animals (febrile state, meningitis, endometritis, hepatopathy and nephropathy).

Regarding the sex variable, the evaluated articles reported a predominance of male animals (*n* = 155) compared to females (*n* = 95). In this context, the study conducted by Dhanani et al. (2020) stood out for obtaining PK data of ceftriaxone through an experimental model using castrated males. The experiment conducted by Ismail (2005) presented results related to lactating goats to explore ceftriaxone concentrations in blood and its elimination in milk. Additionally, research by Rebuelto et al. (2002) adopted a comparative methodology between both sexes in the canine species.

Analysis of age data reported in 20 articles revealed significant variability, with only the studies conducted by Guerrini et al. (1985) and Goudah (2008) presenting animals of similar age (2–3 years). In some studies, terminology allowed inference of age range, particularly in calves (Maradiya et al. 2010; Corum et al. 2019; Johal and Srivastava 1999; Soback and Ziv 1988) and foals (Ringger et al. 1998). However, some experimental models did not detail animal age, describing only adult (Hakim et al. 1989) or mature animals (Rebuelto et al. 2002), premature animals or those registered by days of life (Corum et al. 2019).

With respect to body weight, discrepancies were identified in 27 analysed articles, while the experiments conducted by Kumar et al. (2010) and Soback and Ziv (1988) did not report the weight of animals used for PK parameter acquisition. Additionally, data demonstrated ceftriaxone administration both alone and in combination with other drugs, compared across 10 PK profiles, including experiments involving ketoprofen or tolfenamic acid (Cetin et al. 2021), Danhong (Zhang et al. 2019), itraconazole (Cavalier et al. 1997) and caffeine (Kwon et al. 1985; Kwon and Bourne 1986). The identification of experimental protocols involving different dosing regimens within the same study, as well as the induction of distinct clinical conditions, broadened the understanding of ceftriaxone PK parameters across various scenarios.

The rigorous analysis developed during the methodological selection stage sought to prioritize studies aligned with the scope of this review, particularly the PK and PD properties of isolated ceftriaxone administered alternative routes of administration constituted the first group of works excluded during screening, such as intraperitoneal application in horses (Campos et al. 2017) and inhalational administration in mice (Valiulin et al. 2022). According to Torrent Rodríguez et al. 2024 (Ofokansi et al. 2007), drug interactions represent a recurrent challenge in clinical practice, particularly due to their capacity to interfere with drug metabolism or treatment safety, so that appropriate management of compound coadministration must consider aspects such as the intensity and duration of these interactions. In terms of the therapeutic efficacy of ceftriaxone, scientific articles that addressed clinical scenarios unrelated to the treatment of infections had to be disregarded from the review.

### 3.3 Assessment of Qualitative Attributes

**FIGURE 2:**
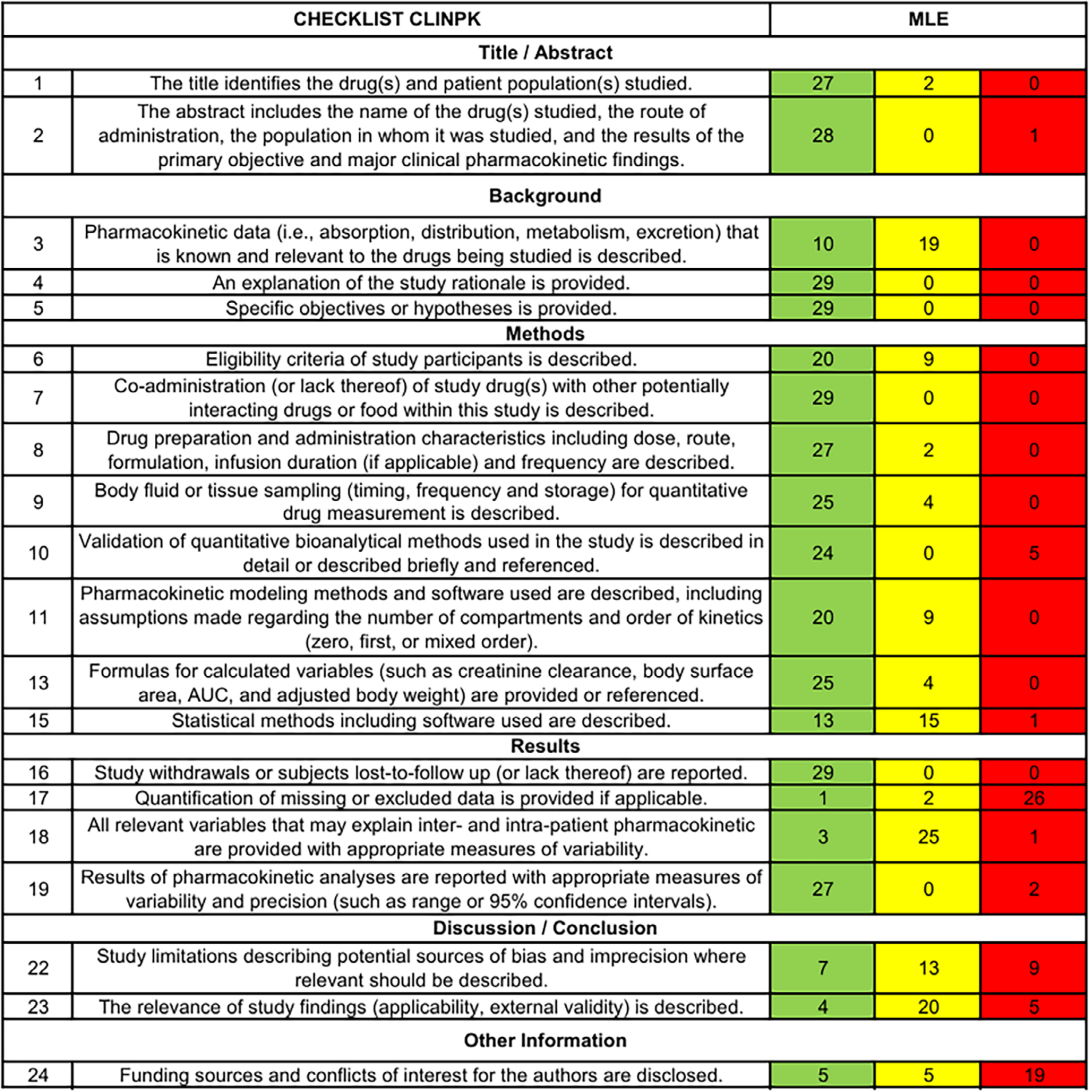
ClinPK analysis of the reviewed reports.

The interpretation of the information evaluated from the investigated article, considering the checklist guiding PK studies, demonstrated results consistent with methodological guidelines, such that they were suitable to be systematized in the EGM, which substantially contributed to the objective analysis of reported evidence (Figure 2). In continuity with the previous stage, the scientific articles submitted to the ClinPK checklist were organized according to the findings evidenced in each analysed item, favouring a clear and consistent interpretation of the respective evidence contained in the studies.

In accordance with the findings evidenced in the checklist of the articles included in the review, important information linked to specific questions was assessed, such as the understanding of the justifications and specific objectives of the studies (Questions 4 and 5), which aimed at the efficacy of ceftriaxone against microorganisms causing injuries in different animal species, as well as how this performance could be achieved through the perception of PK of the compound alone or under coadministration. Additionally, aspects related to the methodology addressed in the articles indicated that single or combined administration of ceftriaxone occurred in all studies (Question 7), and no potential interactions with food provided to the animals were identified, since compound administration was performed intravenously. Subsequently, the evaluation of animal eligibility criteria (Question 6) revealed substantial information for baseline data analysis; however, the findings identified 9 studies that demonstrated partial availability of information regarding the investigated species, particularly data concerning the number of animals, sex, age and weight.

The findings regarding the protocols for handling and administration of ceftriaxone (Question 8) were clearly delineated in the publications, except for the studies by Kovar et al. (1997) and Lutsar et al. (1997), which provided incomplete data due to the absence of specification of the ceftriaxone formulation administered. In addition, the description of blood collection and storage procedures (Question 9) indicates limitations in 4 studies, particularly regarding the detailing of the preservation techniques and the frequency at which collections were performed, whether daily or weekly. Meanwhile, findings related to PK modelling and analysis software (Question 11) revealed omissions of information in 9 studies, including the research by Cavalier et al. (1997) and Ringger et al. (1996), which showed the absence of a description regarding kinetic order and the number of compartments, a condition that may be attributed to the fact that such data were not recurrent requirements among scientific journals at the time. Subsequently, the results concerning mathematical procedures (Question 13) demonstrated that PK parameter modelling in the studies was conducted with samples limited to small groups of animals, thus promoting the adoption of mean and SD values of PK parameters without exploring sources of interindividual variability through covariates.

Regarding the evaluation of the results reported in the articles, it became evident that the PK variables explored (Question 18) indicated relevant contributions to this review, especially in studies reporting the influence of the animals’ clinical conditions on ceftriaxone parameters, such as the studies by Sar et al. (2008) and Ranjan et al. (2011). In addition, the analysis of precision measures (Question 19) found that only the studies by Kwon and Bourne (1986), Rebuelto et al. (2002) and Ringger et al. (1996) reported only mean values of certain PK parameters and presented an absence of SD.

### 3.4 Pharmacokinetic Parameters of Ceftriaxone in Animals

In alignment with the previously proposed methodological approach, the evaluation of the extracted parameters demonstrated consistency among the included studies regarding the adoption of dosing protocols for intravenously administered ceftriaxone. Among the observed findings, 19 studies implemented similar dosage regimens, such as 10 mg kg^−1^ (27.59%), 25 mg kg^−1^ (10.34%), 50 mg kg^−1^ (17.54%) and 100 mg kg^−1^ (10.34%). The study by Lutsar et al. (1997), however, investigated high doses of the compound in guinea pigs (150, 200, 300, 400 mg kg^−1^), aiming to determine the appropriate therapeutic regimen for the treatment of pneumococcal meningitis induced by *S. pneumoniae*.

Continuing the evaluation of the protocols recorded in the articles selected for this review, the studies by Cetin et al. (2021) and Dhanani et al. (2020) presented data related to isolated ceftriaxone at a dose of 40 mg kg^−1^, compared with coadministration with 3 mg kg^−1^ of ketoprofen, 2 mg kg^−1^ of tolfenamic acid (88 mg) and 15 mg kg^−1^ of ertapenem (645.75 mg). Meanwhile, the study by Cavalier et al. (1997) investigated a 36‐day protocol consisting of oral administration of isolated ceftriaxone (1 g) at 12‐h intervals for a period of 5 days, a consecutive 10‐day interval without providing medications to the animals, and finally a bolus administration of ceftriaxone (1 g). The studies by Kwon et al. (1985) and Kwon and Bourne (1986) reported results referring to doses of the single compound (30 and 100 mg kg^−1^) and in association with caffeine (5, 10 and 20 mg kg^−1^). In contrast, Zhang et al. (2019) reported results referring to the isolated dose of ceftriaxone (90 mg kg^−1^ day^−1^), prepared in 0.9% saline solution, as well as its association with different volumes of Danhong (2.19 mL; 15.33 and 30.66 mL), administered at three distinct time windows in aged rats.

In accordance with the previously processed results, the availability of PK parameters of interest across 52 PK profiles in the articles presented substantial data, although in certain evaluated studies, it proved to be limited, particularly regarding values associated with the parameters AUC_0‐t_ (23 articles), AUC_0‐∞_ (5 articles), Cl (Lutsar et al. 1997), Kel or β (11 articles), FU (21 articles), T_1/2_ (Dhanani et al. 2020), V_d_ (3 articles) and α (18 articles). Within this same context, limitations were observed concerning studies that documented SD values of PK parameters, with only mean values identified in some reports, as in the findings described by Kwon and Bourne (1986), Ringger et al. (1996) and Rebuelto et al. (2002).

The PK characteristics of ceftriaxone enabled the development of studies in different compartmental models, and the findings identified 2 studies using a one‐compartment model, 13 studies using a two‐compartment model, and 3 studies using a three‐compartment model (Table 4). Although this evaluation of the articles included such variations in experimental design, it was observed that 6 studies employed noncompartmental models, while 4 studies did not report the modelling structure. In the study conducted by Kwon and Bourne (1986), estimates compatible with a multicompartmental model were described, although the exact number of compartments addressed was not detailed. On the other hand, the study provided a physiologically based schematic representation, highlighting the trajectories of elimination constants and the transfer of ceftriaxone from plasma to peripheral tissue compartments of each organ weighted in the experiment.

**TABLE 4:** Systematization of the compartmental models from the articles selected.

Modelling	References	Expected parameters	Species
One compartment	Lutsar et al. (1997)	Dose, Cl and V_d_	New Zealand White rabbits
	Sar et al. (2008)		Goats Black Bengal
Two compartment	Albarellos et al. (2007)	Dose, Cl, V_1_ and V_2_	Cats mixed breeds
	Gardner and Aucoin (1994)		Mixed breeds mares
	Gohil et al. (2009)		Bubalus bubalis
	Goudah et al. (2006)		Ewes
	Goudah (2008)		Camelus Dromedarius
	Guerrini et al. (1985)		Merino ewes
	Hakim et al. (1989)		Wistar rats
	Ismail (2005)		Goat
	Kovar et al. (1997)		Wistar rats
	Kwon et al. (1985)		Australian original white rabbits
	Maradiya et al. (2010)		Crossbred calves
	Matsui et al. (1984)		Beagle e Rhesus
	Ranjan et al. (2011)		Chhotanagpuri sheep
	Soback and Ziv (1988)		Calves (Israeli‐Friesian)
Three compartment	Dardi et al. (2005)	Dose, Cl, V_1_, V_2_ and V_3_	Buffalo calves
	Johal and Srivastava (1999)		Crossbred calves
	Kumar et al. (2010)		Crossbred Cows

*Note*: V_1_: volume of distribution of the central compartment; V_2_: volume of distribution of the peripheral compartment; V_3_: volume of distribution of the secondary peripheral compartment; Q: intercompartmental clearance; Q_1_: intercompartmental clearance between the central and peripheral compartments; Q_1_: intercompartmental clearance between the central and the second peripheral compartments.

Following the aspects discussed, experiments that addressed multicompartmental models exhibited limitations regarding the availability of parameters referring to the volume of distribution of ceftriaxone for the central compartment (V_1_) and the peripheral compartment (V_2_). Although the findings revealed that 7 studies recorded values related to the V_1_ parameter, the results for V_2_ were limited to the articles, which reported the following: 1.3516 ± 0.124 in dogs and 0.11424 ± 0.2184 in monkeys (Matsui et al. 1984), 0.4205 ± 0.3074 for isolated ceftriaxone, 0.0899 ± 0.0232 for ceftriaxone coadministration with caffeine (5 mg) and 0.1044 ± 0.0203 for ceftriaxone coadministration with caffeine (10 mg) (Kwon et al. 1985).

The physicochemical factors inherent to the behaviour of antimicrobials, with emphasis on cephalosporins, combined with the biological characteristics of patients, play a determining role in modulating the ADMET processes (absorption, distribution, metabolism, excretion and toxicity) of the compound (Kauss et al. 2019; Kar et al. 2025). In light of this theoretical framework, the data synthesized in this review demonstrated pharmacokinetic aspects of intravenous administration of ceftriaxone that differed significantly among the species investigated in the selected articles (Table 3).

In the conceptual exposition by Curry and Whelpton 2022 (Torrent Rodríguez et al. 2024), the parameters AUC_0‐t_ or AUC_0‐∞_ are recognized as assuming a central role in the interpretation of systematic kinetics, as they constitute a metric capable of quantifying drug entry and elimination, with applicability in evaluating patient exposure to the compound of interest. From this perspective, the synthesis of the articles included in this review revealed that fever induced in calves reflected lower AUC_0‐∞_ values (25.2 ± 1.97 µg h mL^−1^), as reported by Dardi et al. (2005), whereas higher values (32.63 ± 1.47 µg h mL^−1^) of this parameter were described by Johal and Srivastava (1999) in healthy calves, despite certain similarities in the pharmacokinetic design of both studies, particularly the three‐compartment modelling. Although different cephalosporins and species were administered, the study by Sagar et al. 2021 (Alikhani et al. 2025) demonstrated that fever induction cannot promote significant alterations in the pharmacokinetic parameters of cefquinome, a third‐generation cephalosporin administered intravenously in goats. Meanwhile, the research by Kwon et al. (1985) associated with the increase in AUC_0‐∞_ (949 ± 229 µg h mL^−1^) of ceftriaxone in rabbits with concomitant administration of caffeine, an interpretation later discussed by Woziwodzka et al. 2022 (Taverne et al. 2016) in the context of the potential adjuvant role of caffeine for third‐generation cephalosporins and its influence on rapid drug elimination. However, the results presented in the study by Kwon and Bourne (1986) revealed a significant reduction in C_max_ (36.6%) and AUC (54.5%) of ceftriaxone in the rat brain due to cerebral hemodynamic alterations attributed to caffeine. Complementarily, compiled results referring to AUC_0‐t_ demonstrated wide variations according to the clinical condition of the animals, reaching minimum values (1.393 ± 273 µg h mL^−1^) in rats with meningitis (Lutsar et al. 1997) and maximum values (570,000 ± 144,000 µg h mL^−1^) in healthy rats (Hakim et al. 1989). Nevertheless, the study by Hertzsch and Richter 2022 (Curry and Whelpton 2022) demonstrated greater ceftriaxone exposure in the cerebrospinal fluid (CSF) of dogs with meningitis, reflected by a higher CSF AUC/plasma AUC ratio (0.224) in neurologically compromised dogs, contrasting with the low ratio value (0.01) recorded in healthy dogs.

The combined analysis of ceftriaxone V_d_ values extracted from the studies included in this review demonstrated that the lowest value of the parameter (0.096 ± 0.012 L kg^−1^) was recorded in rats (Hakim et al. 1989), whereas the highest V_d_ value (1.55 ± 0.52 L kg^−1^) was associated with cows (Kumar et al. 2010). As reported in the literature and exploratory research, physiological factors associated with animal body size may influence drug distribution kinetics, presenting an allometric relationship with blood flow and compound circulation time, culminating in faster distribution rates in smaller species (Sagar et al. 2021; Woziwodzka et al. 2022; Hertzsch and Richter 2022). In addition, the study by Kumar et al. (2010) indicated that inflammatory conditions may influence drug disposition, as elevated V_d_ values of ceftriaxone were recorded. Properties of ceftriaxone are relevant in limiting compound distribution through lipophilic tissues due to its high polarity, elevated molecular weight and strong plasma protein binding within the vascular compartment (Li et al. 2015; Odival Cezar 2011).

Within the discussions raised by Ahmed et al. 2025 (Sharma and McNeill 2009), the impact of analysing plasma protein binding for β‐lactam antibiotics was emphasized, as this parameter has proven to be less predictable and highly variable among species. Although the availability of this information was limited in the articles included in the review, synthesis of the results demonstrated relevant differences among the evaluated models. It was verified that the lowest percentages of minimum FU of ceftriaxone (5.6%–32.8%) were associated with a study conducted in rats (Kwon and Bourne 1986), whereas the highest values of this parameter (80%–71%) were reported in turtles (Mapongpeng et al. 2019). In parallel, exploratory research has already reported broad variability of ceftriaxone FU in different animal species, encompassing percentages of 20%–30% in cattle (Johal and Srivastava 1999; Soback and Ziv 1988), 25% in dogs (Santos et al. 2006), 39%–45% in goats (Ismail 2005) and 84%–96% in rats and rabbits (Santos et al. 2006; Wanat 2020). Complementarily, the work by Ahmed et al. (2025) described the analytical relevance of the distinct interaction level of ceftriaxone with types of plasma proteins in animals, presenting a comparison between the intermediate affinity of the compound with α1‐acid glycoprotein (AAG) in bovine and rat plasma, common in infection and inflammatory states, with the high drug binding to plasma albumin in both animal models.

As explored in the studies by Ahmed et al. 2022 (Sharma and McNeill 2009) and Di 2021 (Ahmed et al. 2022), understanding plasma protein binding of antimicrobials is fundamental for interpreting compound disposition in patients, as it modulates central pharmacokinetic processes, directly reflecting on parameters such as T_1/2_, Kel (or β) and Cl. From this perspective, the T_1/2_ data recorded in the selected scientific articles indicated differences between mammals and reptiles, requiring particular attention during parameter evaluation. In this context, the study by Sar et al. (2008) reported minimum T_1/2_ values (0.19 ± 0.002 h) in the experimental goat model, whereas the research by Mapongpeng et al. (2019) recorded prolonged T_1/2_ (5.81 ± 0.7 h) in turtles. As expected, analysis of Kel results indicated inverse behaviour, with low values of 0.12 ± 0.02 h kg^−1^ associated with turtle modelling (Mapongpeng et al. 2019) and higher records of parameters (3.57 ± 0.04 h kg^−1^) in research conducted in goats (Sar et al. 2008). In general, physiological characteristics specific to reptiles, especially the relationship between temperature and metabolic rate, influence the low transfer of body fluids between vascular and extravascular compartments and reduce the compound elimination rate in these species (Mapongpeng et al. 2019; Popick et al. 1987; Granero et al. 1995).

In the final evaluation of the pharmacokinetic profiles of the reviewed studies, wide interspecies variability regarding Cl was identified, with minimum values of 0.0181 ± 0.0351 L h^−1^ kg originating from the experimental design in monkeys (Matsui et al. 1984), whereas maximum values of 1.41 ± 0.24 L h^−1^ kg were observed in goats (Sar et al. 2008). Additionally, the study by Li et al. 2015 (Sagar et al. 2021) pointed out that in comparative pharmacokinetics, drug behaviour among different species encompasses a range of aspects to be considered, such as the impact of feeding habits, promoting low clearance values of an investigated drug in carnivores, intermediate results in omnivores, and elevated rates in herbivores. Finally, the research by Ahmed et al. 2022 (Sharma and McNeill 2009) reinforced that extrapolation of pharmacokinetic results of antimicrobials from animal models remains limited, such as those related to compound elimination in monkeys, due to methodological restrictions associated with low sample representativeness, despite the analytical model approach incorporating body weight and other interspecies physiological covariates.

## 4 Conclusions

The present systematic review demonstrated that intravenously administered ceftriaxone exhibits a pharmacokinetic profile consistent with rapid systemic availability, wide distribution, and a relatively prolonged elimination half‐life across different animal species. However, parameters such as clearance, volume of distribution, and AUC showed high interspecies variability, influenced by physiological factors, clinical status, and coadministration of drugs. Additionally, high plasma protein binding was shown to be a determining factor for systemic persistence of the drug, highlighting the relevance of the free fraction for therapeutic efficacy.

## Author Contributions

**Michel Leandro de Campos**: conceptualization, investigation, funding acquisition, writing – original draft, writing – review and editing, formal analysis, supervision. **Gabriel de Brito Rodrigues**: investigation, writing – original draft. **Paula Maria Fernandes de Vasconcelos**: investigation, writing – original draft, writing – review and editing, methodology.

## Conflicts of Interest

The authors declare no conflicts of interest.

## Data Availability

The data that support the findings of this study are available from the corresponding author upon reasonable request.
